# Effects of wolfberry (*Lycium barbarum*) consumption on the human plasma lipidome and its association with cardiovascular disease risk factors: a randomized controlled trial of middle-aged and older adults

**DOI:** 10.3389/fnut.2024.1258570

**Published:** 2024-02-19

**Authors:** Darel Wee Kiat Toh, Hanzhang Zhou, Amaury Cazenave-Gassiot, Hyungwon Choi, Bo Burla, Anne Katherin Bendt, Markus R. Wenk, Lieng Hsi Ling, Jung Eun Kim

**Affiliations:** ^1^Department of Food Science and Technology, Faculty of Science, National University of Singapore, Singapore, Singapore; ^2^Department of Biochemistry and Precision Medicine TRP, Yong Loo Lin School of Medicine, National University of Singapore, Singapore, Singapore; ^3^Singapore Lipidomics Incubator (SLING), Life Sciences Institute, National University of Singapore, Singapore, Singapore; ^4^Department of Medicine, Yong Loo Lin School of Medicine, National University of Singapore, Singapore, Singapore; ^5^Department of Cardiology, National University Heart Centre, Singapore, Singapore

**Keywords:** antioxidant, cardiovascular disease, healthy diet, lipidomics, lipids, lipoproteins, *Lycium barbarum*

## Abstract

**Background:**

Long-term wolfberry intake as part of a healthy dietary pattern was recognized to have beneficial vascular outcomes. Characterization of the plasma lipidome may further provide comprehensive insights into pathways underlying these cardiovascular protective effects.

**Objective:**

We analyzed the plasma lipidome of subjects who adhered to a healthy dietary pattern either with or without wolfberry and investigated the associations between the plasma lipidomic profile and cardiovascular health-related indicators.

**Methods:**

In this 16-week, parallel design, randomized controlled trial, middle-aged and older adults (*n* = 41) were provided dietary counseling and assigned to either consume or not consume 15 g of wolfberry daily. At baseline and post-intervention, plasma lipidomics was assayed, and its relationships with classical CVD risk factors, vascular health, oxidant burden, carotenoids status, body composition, and anthropometry were examined.

**Results:**

From the plasma lipidome, 427 lipid species from 26 sub-classes were quantified. In the wolfberry and control groups, significant changes were prominent for 27 and 42 lipid species, respectively (*P* < 0.05 with > 0.2-fold change). Fold changes for seven lipid species were also markedly different between the two groups. Examining the relationships between the plasma lipidome and CVD-related risk factors, total cholesterol revealed a marked positive correlation with 13 ceramide species, while HDL-cholesterol which was notably increased with wolfberry consumption showed a positive correlation with 10 phosphatidylcholine species. Oxidant burden, as represented by plasma 8-isoprostanes, was also inversely associated with lipidomic triglycerides and ether-triglycerides (41 species) and directly associated with hexosylceramides (eight species) and sphingomyelins (six species). There were no differential associations with CVD risk detected between groups.

**Conclusion:**

Characteristic alterations to the plasma lipidome were observed with healthy dietary pattern adherence and wolfberry consumption. An examination of these fluctuations suggests potential biochemical mechanisms that may mediate the antioxidant and cardiovascular protective effects of healthy dietary pattern adherence and wolfberry intake. This study was registered at clinicaltrials.gov as NCT0353584.

## 1 Introduction

Cardiovascular diseases (CVD) are a leading cause of global mortality, responsible for ~38 % of non-communicable disease related deaths (1). According to the INTERHEART, a multi-national study which recruited 15,000 coronary heart disease (CHD) cases and a similar number of matched controls, dyslipidemia was the single most important factor predisposing myocardial infarction (2). Identification and control of this and other risk factors have been shown to alleviate future CHD burden (3). However, standard blood lipid-lipoproteins concentrations portray an oversimplified view of human plasma lipids which comprise thousands of structurally and functionally diverse species (4).

A complete characterization of the lipid fingerprint in a biological system with omics technology functions as a powerful platform to holistically investigate interactions between nutrition, metabolism and genotypic variability. This offers a detailed examination on the risks of diseases such as CVD, which are fundamentally linked to lipid dysregulation (5, 6). In a clinical setting, a global characterization of lipid species can potentially provide insights into biochemical and metabolic pathways underlying any observed changes to cardiovascular health-related biomarkers, and also be potentially used to more closely monitor intervention response (7).

Among the lifestyle modifications targeted at lowering CVD risk, a large body of scientific literature substantiates the importance of diet and nutrition (8, 9). Healthy dietary patterns (HDPs), broadly characterized by a balanced consumption of fruits, vegetables, wholegrains, and protein-rich foods serve as a practical and sustainable approach to maintain good cardiovascular health (10). Moreover, this may be augmented by the consumption of specific foods with postulated cardiovascular protective benefits. One such example is the wolfberry (*Lycium barbarum*), a well-known food consumed historically in Asia that contains a variety of bioactive constituents (including carotenoids, phenolics, vitamin C precursor and prebiotic polysaccharides) that may modulate CVD risk (11–13).

In animal models, wolfberry had been evidenced to inhibit lipogenic genes (e.g., acetyl-CoA carboxylase, fatty acid synthase, elongation of very long chain fatty acids protein 6, and diglyceride acyltransferase) and lower circulating triacylglyceride (TG) levels (14, 15). Additionally, it may promote the expression of cholesterol 7-alpha-hydroxylase, the rate-limiting enzyme that catalyzes the production of bile salts from cholesterol (16). This is supported by wolfberry-based clinical trials which reported favorable changes to blood lipid-lipoproteins following a 3 months intervention with *L. barbarum* polysaccharides (100 mg/kg body weight daily) and a 45-day intervention with whole wolfberry (14 g dried fruit per day) (17–19). Previously, we demonstrated notable improvements in HDL cholesterol (HDL-C) concentration (20) and oxidative stress status (21) following the consumption of 15 g/day whole wolfberry with a HDP for 16 weeks. Nevertheless, limited studies examined these blood lipid changes in detail and the potential mechanisms that underlie the observed cardiovascular protective benefits.

The objective of this study was to characterize alterations to the plasma lipidome of middle-aged and older adults who completed the 16-week randomized controlled trial (RCT) of wolfberry consumption. Associations between lipidomic species, with the corresponding changes in CVD-related indicators (including lipid-lipoproteins profile, blood pressure, vascular health, oxidative stress status, and body composition) were further evaluated to explore the potential mechanisms that underlie the cardiovascular protective effects of wolfberry.

## 2 Materials and methods

### 2.1 Study design and subjects

Study design specifics and subject recruitment criteria for the present 16-week, parallel design, single-blind (investigator) RCT had been described previously (20). The protocol was approved by the National Healthcare Group Domain Specific Review Board and was registered in clinicaltrials.gov as NCT03535844. Informed consent was provided by all subjects prior to participation and the study was conducted between July 2018 and October 2019 at the National University of Singapore and National University Hospital.

**Figure 1:**
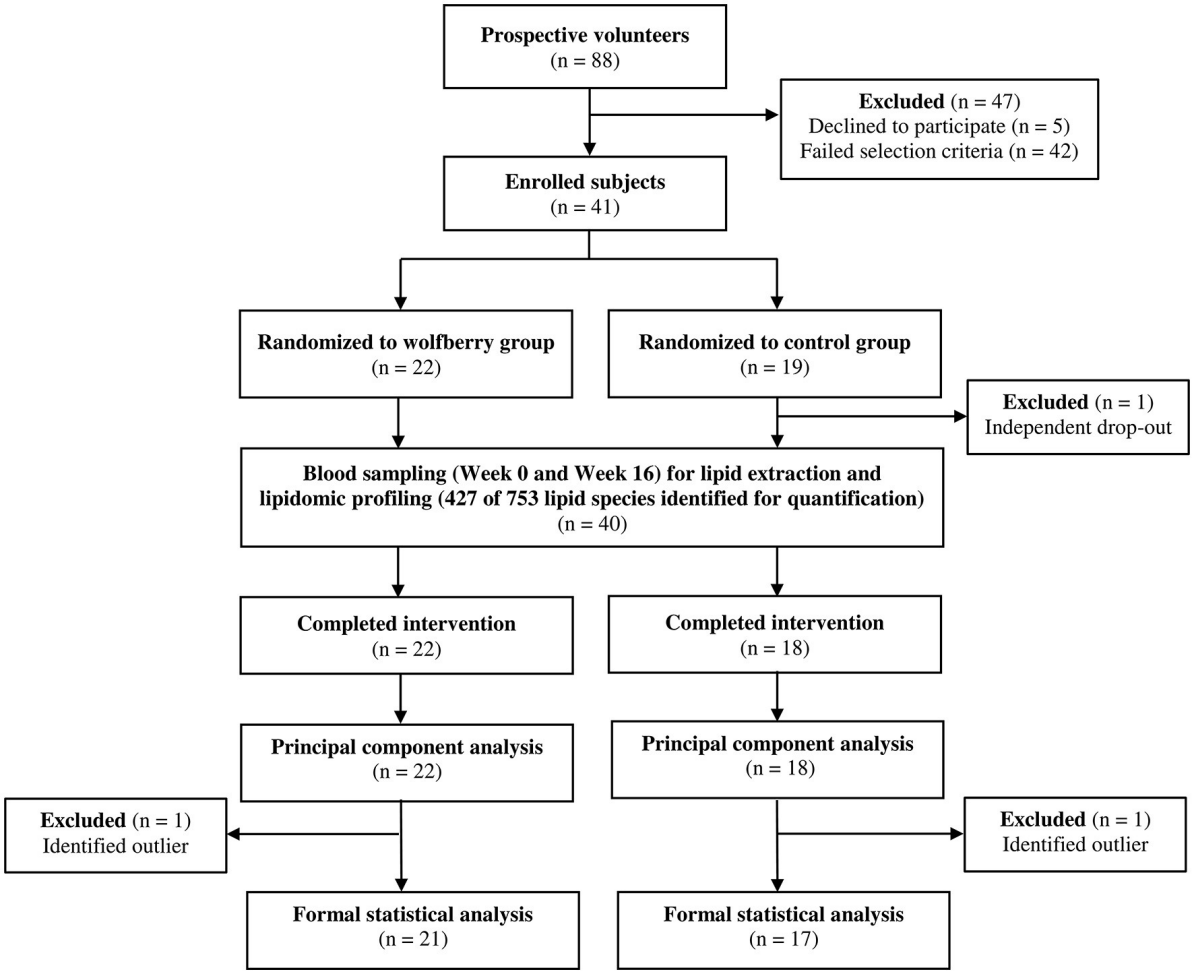
Participant flow diagram.

Apparently healthy, Singaporean men and women (*n* = 41) were recruited by the research team based on a set of inclusion and exclusion criteria established *a priori* (20), and randomly assigned (STATA/MP version 13; StataCorp LP) to either the wolfberry (*n* = 22) or control (*n* = 19) groups by a researcher who was independent of the screening process (Figure 1). During the intervention duration, one subject from the control group withdrew due to reasons that were study independent. Plasma lipidome characterization was conducted for all subjects who completed the full study duration (*n* = 40) although data analyses and interpretations were limited to 21 and 17 from the wolfberry and control groups respectively after the omission of outliers who were identified as clear anomalies via principal component analysis (PCA).

During the first visit, subjects were provided with an instructions sheet, as well as a one-to-one dietary counseling with a research nutritionist to adhere to HDP recommendations by the Singapore Health Promotion Board—“My Healthy Plate (MHP).” Compliance was broadly defined by a balanced consumption of wholegrains (4–6 servings/day), fruits (2 servings/day), vegetables (2 servings/day), and unprocessed protein-rich foods (3 servings/day including 1 serving from a calcium-rich source) (22). The wolfberry group additionally received 500 g dried wolfberries (Ning Xia, China; purchased from Eu Yan Sang Pte. Ltd., Singapore) every 4 weeks, along with specific instructions to cook (e.g., by steaming or heating in hot water) and consume 15 g dried wolfberry per day (both the fruit and liquid used for preparation) as part of a mixed-meal. The dosage was determined based on prior evidence which reported marked effects on plasma zeaxanthin concentrations and biomarkers of oxidative stress following treatment at 15 g wolfberry daily (18, 23).

To optimize MHP adherence, dietary counseling sessions were conducted every 4 weeks (Weeks 0, 4, 8, and 12). Based on 3-day food records completed, each subject was provided with individually tailored instructions that addressed on gaps in dietary compliance. In addition, wolfberry intake was objectively examined via an assay of plasma zeaxanthin concentrations. The extraction and high-performance liquid chromatography (HPLC) methodology used for plasma carotenoids analysis was as previously described (24).

### 2.2 Blood sampling and extraction

At weeks 0 and 16, blood was sampled by intravenous cannulation into K2EDTA-coated vacutainers (Becton Dickinson). After centrifugation (15 min at 3,000 × *g*, 4°C) plasma samples were aliquoted and stored at – 80°C prior to analysis.

The single-phase lipid extraction and HPLC/mass spectrometry (MS)-MS analytical protocol was adapted from Gao et al. (25) with modifications. Plasma (10 μL) was vortexed with 190 μL butanol:methanol (1:1, by volume) spiked with internal standards (detailed in Supplementary Table 1) and sonicated in an ice water bath for 30 min. The mixture was then centrifuged (20,800 × *g* for 10 min at 4°C) with the supernatant collected for analysis. Solvents used for extraction were HPLC grade, from Merck Millipore.

### 2.3 Plasma lipidome profiling

Extracted lipids were characterized by HPLC (Agilent 1290) fitted with a reversed-phase Agilent ZORBAX Eclipse Plus (C_18_) column (50 mm × 2.1 mm) and a pair of mobile phases (eluent A (acetonitrile:water; 2:3 by volume, with 10 mmol/L ammonium formate) and eluent B (acetonitrile:isopropanol; 1:9 by volume, with 10 mmol/L ammonium formate). The programmed flow, delivered at 0.4 mL/min, was conducted at 25°C with the following gradient: eluent B = 20% held for 2 min, linear increase to eluent B = 60% at 7 min for a 2 min hold, followed by a rapid increase to eluent B = 100% at 9.1 min before re-equilibration to starting conditions at 10.8 min. The oven was maintained at 40°C.

The HPLC system was connected to an Agilent 6460 triple quadrupole tandem MS for a targeted quantification of lipid species using dynamic multiple reaction monitoring mode. The conditions used were as follows: capillary voltage, 3,500 V; drying gas, 17 L/min at 150°C; sheath gas, 10 L/min at 200°C; and nitrogen gas nebulizer at 20 psi.

Raw data were processed with MassHunter QqQ quantitative software (version B.08; Agilent). The retention times coupled with MRM precursor and product ion transitions were used for the identification of lipid species. The relative abundance of each lipid species was calculated via normalization against the respective internal standards (Supplementary Table 1). Batch quality controls (pooled plasma samples separately extracted) and technical quality controls (pooled plasma lipid extracts) were used to track for analytical fidelity between batches and to ascertain system stability. Lipid species were only evaluated if the following criteria were satisfied: (1) signal:noise > 10:1; (2) absolute area under curve > 500 units; and (3) coefficient of variation < 30% (26). From the original 753 lipid species in the panel, 427 species were quantified. This was broadly classified into one of the following classes: acylcarnitine (15 species), cholesterol ester (CE; eight species), ceramide (Cer; 23 species), fatty acid (three species), hexosylceramide (HexCer; 30 species), sphingomyelin (SM; 29 species), sphingosine 1-phosphate (S1P; four species), monosialodihexosylganglioside (GM3; seven species), phosphatidylcholine (PC; 45 species), ether-PC (PC-O; 21 species), plasmalogen-PC (PC-P; 21 species), lysoPC (LPC; 26 species), ether-LPC (LPC-O; 10 species), plasmalogen-LPC (LPC-P; five species), phosphatidylethanolamine (PE; seven species), ether-PE (PE-O; three species), plasmalogen-PE (PE-P; 11 species), lysoPE (LPE; six species), plasmalogen-LPE (LPE-P; four species), phosphatidylinositol (PI; 10 species), phosphatidylserine (PS; three species), lysoPS (LPS; two species), triacylglyceride (TG; 113 species), ether-TG (TG-O; 18 species), sulfatide (two species), and ubiquinone (one species). The plasma lipidome was the primary outcome of the present research.

### 2.4 Power calculation and statistical analysis

Current sample size estimates were based on the changes in oxidant burden which was the primary outcome of interest for the original RCT as described in the earlier publication (27). While lipidomic data derived sample size estimates would have been more rigorous, the high dimensionality and multicollinearity of the lipidome, coupled with a paucity of relevant clinical data limits a robust power calculation. Nevertheless, data generated at present will serve as useful preliminary data for future clinical research.

Between-group comparisons of baseline characteristics were evaluated using Fisher's exact test and independent *t*-test for categorical and continuous variables, respectively. Lipidomic data were transformed using binary logarithm prior to analysis. Fold changes (FC; from baseline) for the wolfberry and control groups were visualized using PCA and partial least squares-discriminant analysis (PLS-DA). Within and between group differences in the FC of lipid species were assessed using paired *t*-test and Welch's independent *t*-test, respectively.

To examine the associations between lipidomic species and CVD-related outcomes, as well as nutritional/dietary data, pairs of continuously scaled outcome variables and lipid species were fitted to a linear mixed effects model with time (weeks 0 and 16) and intervention (wolfberry and control groups) as fixed effects, and random intercept using linear and non-linear mixed effects models (nlme), R library (28). These outcomes comprised of classical CVD risk factors (i.e., serum lipid-lipoprotein concentrations, plasma glucose, blood pressure, etc.), physiological and serological indicators of vascular health and function (i.e., flow mediated dilation, carotid intima-media thickness, plasma nitrate/nitrite, endothelial progenitor cell count, etc.), biomarkers of oxidative stress, skin and plasma carotenoids status, anthropometry, and body composition. Prior to model fitting, both the outcome variable and lipid concentration data were standardized (mean 0, standard deviation 1). The statistical significance values reported by individual linear mixed effects models were compiled and multiple testing correction was applied to control the overall type I error (29). Given the modest total number of statistically significant associations, all pairs with *Q*-value < 0.2 were considered as significant findings. For heatmap visualization, rows and columns were subjected to hierarchical clustering with Euclidean distance metric and average linkage function, using the following data as the input: –log_10_(*P*-value) × sign(regression coefficients).

Statistical analyses (two-tailed, with a statistical significance of 0.05) were performed on Rstudio (version 1.3.959) and STATA/MP (version 13, StataCorp LP). For the identification of more pronounced changes within the human plasma lipidome, a minimum 0.2-fold difference was set as an additional criterion to define significance among lipid species. Data are presented as means ± SD unless otherwise stated.

## 3 Results

### 3.1 Subjects

Subjects examined were middle-aged and older adults (55 ± 4 y) comprising 29 females and nine males. Baseline characteristics (Table 1) indicated an apparently healthy population of non-smokers, with BMI, clinic blood pressure, fasting plasma glucose and the classical serum lipid-lipoproteins concentrations [TG, total cholesterol (TC), LDL-cholesterol (LDL-C), and HDL-C] all within normal ranges stipulated by the American Heart Association, American Diabetes Association and Ministry of Health, Singapore (30–34). This aligns with the Framingham vascular age: 47 ± 11 y, which was ~8 years younger than the population's physiological age (35). There were no restrictions on chronic prescription medication, as long as stable use (>5 y) was reported. With reference to these characteristics, there were no differences between the wolfberry and control groups. No adverse events were reported throughout the study duration.

**Table 1:** Baseline characteristics of the study population.

**Characteristics**	**Control (*n* = 17)**	**Wolfberry (*n* = 21)**	**Combined (*n* = 38)**	***P* ^a^**
Sex	6 M 11 F	3 M 18 F	9 M 29 F	0.25
Age (y)	55 ± 4	56 ± 4	55 ± 4	0.32
BMI (kg/m^2^)	22.7 ± 2.5	22.7 ± 3.8	22.7 ± 3.2	1.00
Waist circumference (cm)	80.0 ± 7.0	80.1 ± 9.7	80.1 ± 8.5	0.97
Triglycerides (mmol/L)	1.2 ± 0.5	1.1 ± 0.5	1.2 ± 0.5	0.65
Total cholesterol (mmol/L)	5.4 ± 1.2	5.2 ± 0.9	5.3 ± 1.0	0.41
LDL-cholesterol (mmol/L)	3.3 ± 1.1	3.1 ± 0.6	3.2 ± 0.9	0.38
HDL-cholesterol (mmol/L)	1.6 ± 0.4	1.6 ± 0.5	1.6 ± 0.4	0.99
Fasting glucose (mmol/L)	5.0 ± 0.6	4.8 ± 0.7	4.9 ± 0.6	0.34
Systolic blood pressure (mmHg)	106 ± 13	114 ± 19	110 ± 17	0.13
Diastolic blood pressure (mmHg)	69 ± 9	73 ± 12	71 ± 11	0.24
Framingham CVD risk (%)	5.4 ± 4.3	5.4 ± 5.0	5.4 ± 4.5	0.99
Vascular age (y)	45 ± 10	49 ± 11	47 ± 11	0.39

^a^Between group differences examined by Fisher's exact test or independent t-test for categorical and continuous characteristic variables, respectively.

### 3.2 Plasma lipidomic profiling

The PCA plot was obtained based on the FC of all 427 quantified lipid species and was used to characterize the dataset and identify potential outliers as depicted in Supplementary Figure 1. Although there were no distinct discriminations observed between the wolfberry and control groups, further visualization using a supervised clustering algorithm (PLS-DA) for the FC between the wolfberry and control groups showed a separation of the plasma lipidome with and without wolfberry consumption (Supplementary Figure 1).

**Figure 2:**
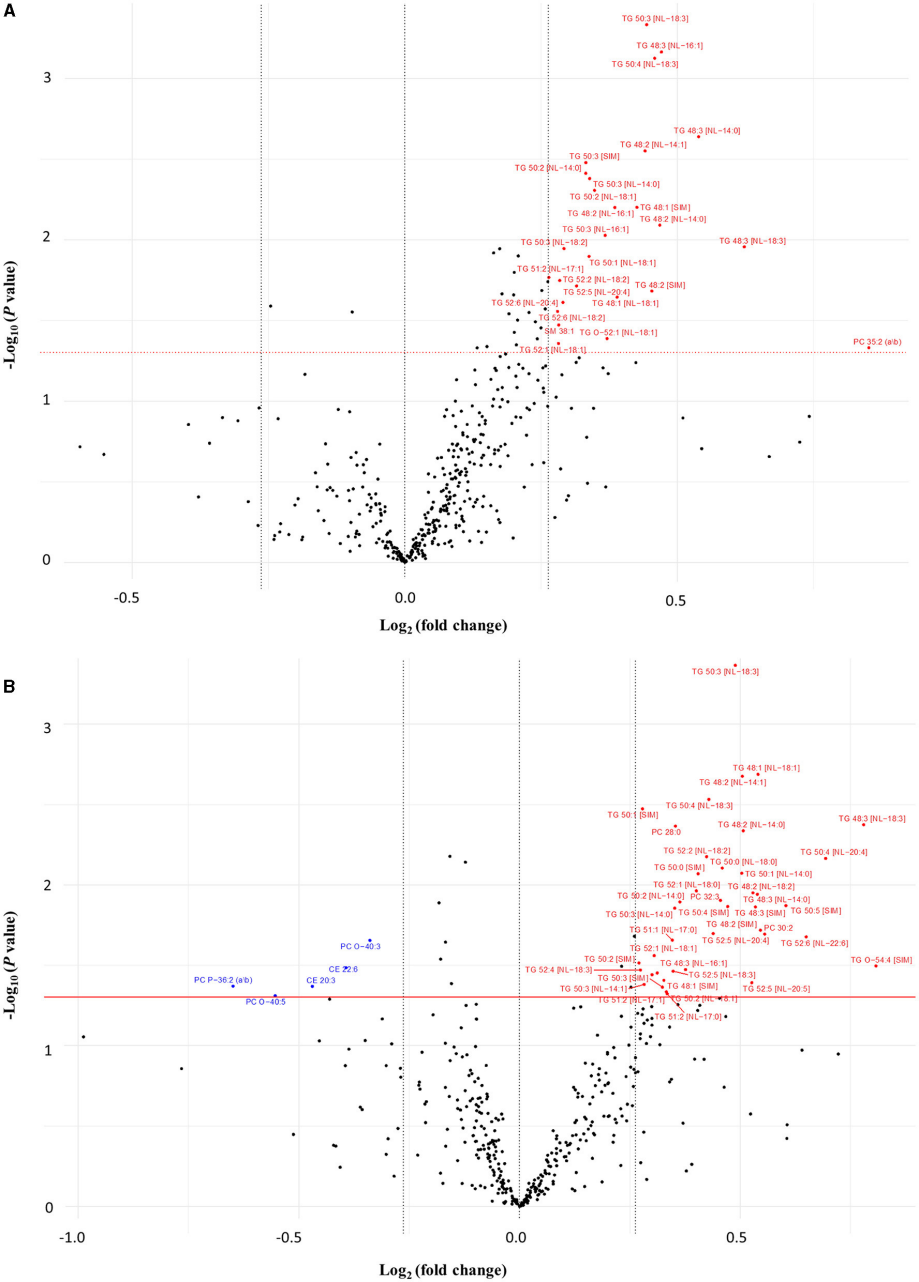
Volcano plot comparing the fold changes of individual lipid species between week 16 and baseline for **(A)** wolfberry group and **(B)** control group. Species significantly increased (in red) and decreased (in blue) based on paired *t*-tests (*P* < 0.05) with a minimum 0.2-fold difference. CE, cholesterol ester; Hex1Cer, monohexosylceramide; ns, no significant difference; PC, phosphatidylcholine; PC-O, ether-phosphatidylcholine; PC-P, plasmalogen-phosphatidylcholine; SM, sphingomyelin; TG, triglyceride; TG-O, ether-triglyceride.

After the 16-week intervention period, significant within-group changes were observed amongst several lipid species (visualized by volcano plots depicted in Figure 2). Concentrations of 24 and 33 TG species were markedly higher at week 16 in the wolfberry and control groups, respectively compared to the baseline. Among the other lipid species, the plasma concentrations of PCs 28:0, 30:2, 32:3, and TG-O 54:4 were increased while those of CEs 20:3 and 22:6, PC-Os 40:3, and 40:5, and PC-P 36:2 were decreased in the control group. In the wolfberry group, prominent positive FC were reported for PC 35:2, SM 38:1, and TG-O 52:1.

**Figure 3:**
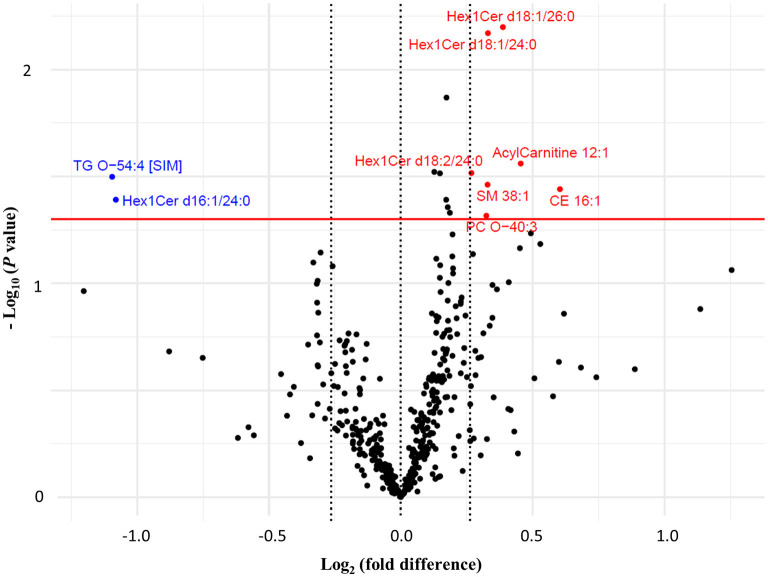
Volcano plot comparing the fold changes of individual lipid species between the wolfberry group and control group. Species with higher fold change value in wolfberry group (in red) and control group (in blue) defined based on Welch's independent *t*-tests (*P* < 0.05) with a minimum 0.2-fold difference. CE, cholesterol ester; Hex1Cer, monohexosylceramide; ns, no significant difference; PC, phosphatidylcholine; PC-O, ether-phosphatidylcholine; PC-P, plasmalogen-phosphatidylcholine; SM, sphingomyelin; TG, triglyceride; TG-O, ether-triglyceride.

Between groups, FC of seven lipid species were significantly different at week 16 (Figure 3). These included lipid species that were characteristically higher in either the wolfberry [acylcarnitine 12:1, CE 16:1, monohexosylceramides (Hex1Cers) d18:1/24:0, d18:1/26:0 and d18:2/24:0, PC-O 40:3 and SM 38:1] or control groups (Hex1Cer d16:1/24:0 and TG-O 54:4; Figure 3). Individual alterations to lipid species with either significant within- or between-group differences are more comprehensively illustrated in Supplementary Figure 2 (with the exception of TG species). According to the loading weights for PLS-DA component 1 which explained 9 % of variance (Supplementary Figure 3), the deduced clustering aligned with findings from the above statistical tests, identifying a similar selection of discriminating lipid species between groups.

### 3.3 Relationships between the plasma lipidome and CVD-related risk indicators and nutrition/diet

Associations between the plasma lipidomic changes and the corresponding effects on a panel of CVD-related risk indicators (*n* = 47) and nutrition/dietary information (*n* = 35) were studied (20, 21). Main effects (i.e., between lipid species and outcome of interest) are summarized in heat maps (Supplementary Figure 4) with the main effect size and Q values of significant pairs additionally detailed in Supplementary Table 2. A majority of the TG species (*n* = 103) exhibited positive main effects with serum TG. Among the remaining serum lipoproteins, positive associations were prominent between TC and Cer (*n* = 13), PC (*n* = 5), and SM (*n* = 3); LDL-C and Cer (*n* = 3) as well as HDL-C and PC (*n* = 10), Cer (*n* = 3), and SM (*n* = 2). Diastolic blood pressure showed negative main effects with some PC species (*n* = 5).

Changes in oxidative stress status (i.e., plasma 8-isoprostanes) were negatively correlated with HexCer (*n* = 8) and SM (*n* = 6) although positive associations were observed with TG and TG-O (*n* = 41). Plasma zeaxanthin on the other hand reflected positive main effects with LPC (*n* = 8). There was however a lack of interaction effects across all of the outcomes which revealed an absence of differential associations between the wolfberry and control groups (Supplementary Figure 5).

## 4 Discussion

The present RCT characterized the alterations to the plasma lipidome of middle-aged and older adults after a 16-week HDP adherence either with or without wolfberry consumption. Significant changes were observed for 50 lipid species, across both groups. Independent of wolfberry intake, these changes were likely a consequence of compliance to the HDP. Between groups, the plasma lipidome showed modest differences albeit with a characteristic reduction in PC-O 40:3 and increase in SM 38:1 that may be modulated by the consumption of wolfberry. Alterations to the lipidome were also found to be significantly associated with several CVD-related risk indicators and biomarkers. This was represented by main effects for HDL-C (with PC, Cer, and SM), plasma 8-isprostanes (with TG, TG-O, HexCer, and SM) and plasma zeaxanthin (with LPC).

The increases in concentrations of several TG species, evident across both groups, were likely contributed by dietary changes following HDP adherence. Dietary alterations, especially to the type of fat consumed, may elicit a direct effect on the fatty acid profile of circulating TG (36). Given the pronounced dietary modifications (i.e., including changes to the sources of dietary fats and oils consumed) after dietary counseling (20), the type of TG species enriched may thus be consequent of the change and diversification in dietary choices. However, pooled concentrations of all TG species from the lipidome (change wolfberry group: −15 ± 26 μmol/L, *P* = 0.55; control: 9 ± 13 μmol/L, *P* = 0.47) continued to mirror changes that were reported for serum TG (20). This accorded with the significant main effects that were identified between lipidomic TG species with serum TG.

Previously, we observed that the addition of wolfberry to a HDP can increase serum HDL-C concentrations and lower long-term CVD risk (20). Among the other lipid classes, PC-related species (i.e., PC, PC-O, and PC-P) in particular were positively correlated to HDL-C across the study population. As the key phospholipid instrumental for HDL assembly, an enrichment of PC in plasma promotes apolipoprotein A1 redistribution which enhances nascent HDL formation and thus, serum HDL-C concentrations (37–39). While the increases in PC were not group-specific, those characteristically raised with wolfberry intake trended toward species that had generally longer carbon chains. This corresponds to the main effects observed between HDL-C and PC which likewise revealed more prominent associations among middle- and long-chain PCs (C:18 and longer). While it may be postulated that the longer fatty acid chains enhance HDL membrane fluidity and better preserves circulating concentrations, the underlying mechanisms remain unclear (39).

A sphingolipid characteristically raised with wolfberry consumption is SM 38:1. According to a previous *in vitro* study, wolfberry was reported to exhibit acid sphingomyelinase (ASMase) inhibitory activity, which hinders the hydrolysis of SM to Cer that are deleterious to cardiovascular health (40, 41). Moreover, ASMase may impair peripheral blood flow and vascular function, as well as contribute to atherosclerotic progression via sub-endothelial lipoprotein aggregation (42). Nevertheless, further investigation may be warranted since the observed effect was specific to a single SM species.

Plasma SM has however also been identified to be an independent risk factor of CHD although fatty acid chain length may again be a key factor for consideration (43). In the Cardiovascular Health Study, C:16 SM and Cer species were associated with increased risk of incident heart failure, and C:22 and C:24 sphingolipids with lowered risk, possibly via differential effects on biological processes such as apoptosis, oxidative stress, inflammation and lipotoxicity (44). Similar to PCs, SMs are a major component of HDL and is critical for the maintenance of its structural integrity and reverse cholesterol efflux efficiency (43, 45–47). While this supports the present wolfberry group specific increment in serum HDL-C, there remains an absence of significant associations between these lipid-outcome pairs.

SM was however inversely associated with the production of plasma 8-isoprostanes and a reduced oxidant burden. In many aspects, this is in alignment with earlier literature which also established implicit links between oxidative stress and sphingolipid metabolism. Inverse correlations, for instance, were demonstrated between SM and lipid peroxidation (48, 49). This was postulated to be a consequence of the increased molecular order and fluidity of SM-rich membranes which physically hinders against metal ion and free radical attacks, as well as the propagation of lipid peroxidation (48). Furthermore, Cers were demonstrated to induce reactive oxygen species generation (50, 51). Collectively, this facilitates the formation of oxidized LDL and the creation of pro-atherogenic conditions.

Associations between carotenoids status (i.e., including dietary, plasma, and skin carotenoids) and the plasma lipidome were limited to plasma zeaxanthin concentrations and LPCs specifically. Wolfberry, among all foods, is the richest source of zeaxanthin. As presented previously, zeaxanthin was the only carotenoid to be significantly raised in the wolfberry group at week 16 (21). Although interaction effects were absent (potentially due to the limited sample size for an evaluation of correlations), it is logical to assume that the dietary incorporation of wolfberry may be partly responsible. Unique to wolfberry, ~75% of the total carotenoids exists as zeaxanthin dipalmitate which, contrast to its free form, is apolar and structurally less compact (52). While the micellar enrichment of phospholipids including PC and LPC had been evidenced to regulate carotenoid absorption in the gut (53), the significance of the wolfberry derived zeaxanthin diesters and its link to circulating LPC concentrations warrant further investigations.

The prominent elevations of HexCers (i.e., d18:1/24:0 and d18:1/26:0) in the wolfberry group, contrast to the control which depicted slight reductions indicate an effect potentially mediated by wolfberry intake. However, the clinical significance of these observations in our healthy population remain uncertain. In the ADVANCE trial and Singapore Prospective Study Program, circulating HexCers showed a direct relationship with CVD events and risk markers (26, 54). However, HexCers could also confer indirect protective effects having been reported by the Singapore Prospective Study Program to be negatively associated with incident type 2 diabetes mellitus (55).

Overall, while the plasma lipidome serves as a powerful mode of clinical assessment, the few and irregular between group disparities in lipid species limit a robust evaluation. This is especially true when studying its associations with CVD-related risk indicators due to the potential inadequacies in statistical power. Furthermore, observed variabilities could also be amplified by intrinsic factors that include high intra- and inter-individual variability, age- and sex-based discrepancies in response to the intervention diet, as well as the diversity of dietary changes that can occur, beyond wolfberry intake (56). This leads to proportionately smaller changes when considering individual lipidomic species. For example, HDL-C which was raised in the wolfberry group was likely attributed to a culmination of changes from various phospholipids and sphingolipids within HDL. Thus, while the raised serum HDL-C may be apparent, plasma lipidomic alterations could be subtle and challenging to perceive. This is supported by previous clinical research involving other HDP (e.g., the Mediterranean and Nordic diets) which likewise failed to identify any distinct alterations to the plasma lipidome in spite of their well-documented benefits to cardio-metabolic health (39, 57).

Nevertheless, an analysis of the plasma lipidome allows for the study biological changes with unprecedented detail. The present research deduced characteristic alterations to the plasma lipidome following an adherence to a healthy dietary pattern, and with wolfberry consumption. These fluctuations represent both the cellular and biochemical changes that follow the dietary intervention and illustrate the downstream lipidomic alterations that may mediate the role of wolfberry in raising HDL levels and in maintaining the antioxidant network for CVD prevention.

## Data availability statement

The original contributions presented in the study are included in the article/Supplementary material, further inquiries can be directed to the corresponding author.

## Ethics statement

The studies involving humans were approved by National Healthcare Group Domain Specific Review Board. The studies were conducted in accordance with the local legislation and institutional requirements. The participants provided their written informed consent to participate in this study.

## Author contributions

DT: Conceptualization, Data curation, Formal analysis, Investigation, Methodology, Project administration, Resources, Validation, Visualization, Writing – original draft, Writing – review & editing. HZ: Conceptualization, Data curation, Formal analysis, Investigation, Methodology, Validation, Visualization, Writing – original draft, Writing – review & editing. AC-G: Conceptualization, Data curation, Formal analysis, Investigation, Methodology, Software, Supervision, Validation, Visualization, Writing – review & editing. HC: Data curation, Formal analysis, Investigation, Resources, Software, Visualization, Writing – review & editing. BB: Data curation, Formal analysis, Investigation, Methodology, Software, Visualization, Writing – review & editing. AB: Investigation, Supervision, Writing – review & editing. MW: Conceptualization, Investigation, Supervision, Writing – review & editing. LL: Conceptualization, Methodology, Resources, Supervision, Writing – review & editing. JK: Conceptualization, Formal analysis, Funding acquisition, Investigation, Methodology, Project administration, Resources, Software, Supervision, Visualization, Writing – review & editing.
