# Supplementary material for: Effects of wolfberry (Lycium barbarum) consumption on the human plasma lipidome and its association with cardiovascular disease risk factors: a randomized controlled trial of middle-aged and older adults
Source: Front Nutr. 2024 Feb 19;11:1258570. doi: 10.3389/fnut.2024.1258570 (PMC10909962; doi:10.3389/fnut.2024.1258570)
Supplement: Supplementary file 1 [file Data_Sheet_1.docx]

**Effects of wolfberry (*Lycium barbarum*) consumption on the human plasma lipidome and its association with cardiovascular disease risk factors: a randomized controlled trial of middle-aged and older adults**

Online Supplementary Material

**Supplementary Table 1** Internal standards for the normalization of lipid species in the plasma lipidomic profile

| **Lipid class** | **Internal standards** |
| --- | --- |
| Acylcarnitine | AcylCarnitine 16:0 d3 |
| Cholesterol ester | Cholesterol ester 18:0 d6 |
| Ceramide | Ceramide d18:1/25:0 |
| Diacylglycerol | Triacylglycerol 51:0 |
| GM1 ganglioside | Monohexosylceramide d18:1/12:0 |
| Monohexosylceramide | Monohexosylceramide d18:1/12:0 |
| Dihexosylceramide | Dihexosylceramide d18:1/12:0 |
| Lysophosphotidylcholine | Lysophosphotidylcholine 13:0 |
| Lysoalkylphosphatidylcholine | Lysophosphotidylcholine 13:0 |
| Lysoalkenylphosphatidylcholine | Lysophosphotidylcholine 13:0 |
| Phosphatidylethanolamine | Phosphatidylcholine 26:0 |
| Alkylphosphatidylethanolamine | Phosphatidylcholine 26:0 |
| Alkenylphosphatidylethanolamine | Phosphatidylcholine 26:0 |
| Lysophosphatidylethanolamine | Lysophosphatidylethanolamine 14:0 |
| Lysoalkenylphosphatidylethanolamine | Lysophosphatidylethanolamine 14:0 |
| Phosphatidylinositol | Phosphatidylinositol 25:0 |
| Lysophosphatidylinositol | Phosphatidylcholine 26:0 |
| Phosphatidylcholine | Phosphatidylcholine 26:0 |
| Alkylphosphatidylcholine | Phosphatidylcholine 26:0 |
| Alkenylphosphatidylcholine | Phosphatidylcholine 26:0 |
| Phosphatidylglycerol | Phosphatidylcholine 26:0 |
| Phosphatidylserine | Phosphatidylcholine 26:0 |
| Sphingosine-1-phosphate | Sphingosine-1-phosphate d17:0 |
| Sphingomyelin | Sphingomyelin 30:1 |
| Sphingosine | Sphingomyelin 30:1 |
| Sulfatide | Dihexosylceramide d18:1/12:0 |
| Triacylglycerol | Triacylglycerol 51:0 |


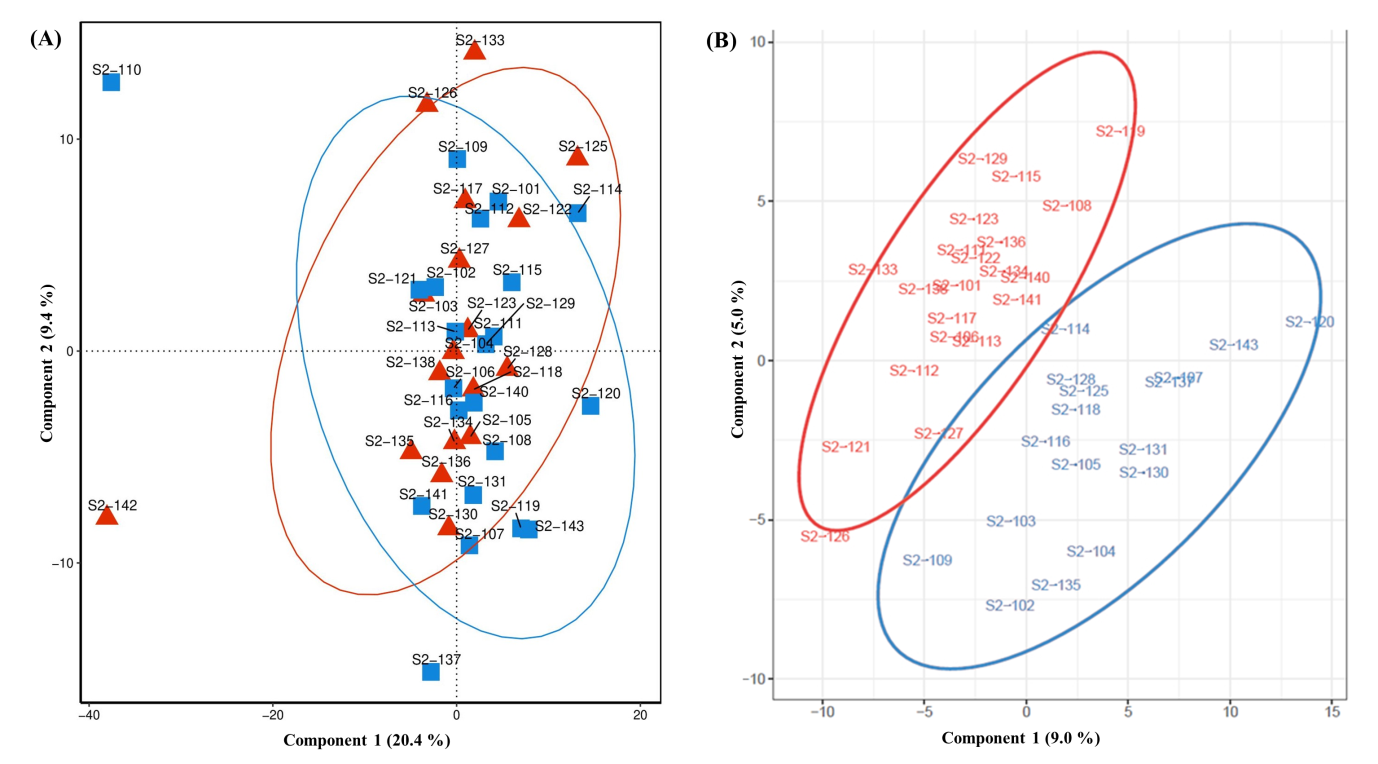


**Supplementary Figure 1** Principal component analysis (A) and least squares-discriminant analysis (B) based on the fold changes of lipid species in the plasma lipidome at week 16. Red (triangles) represent the wolfberry group while blue (squares) represent the control group


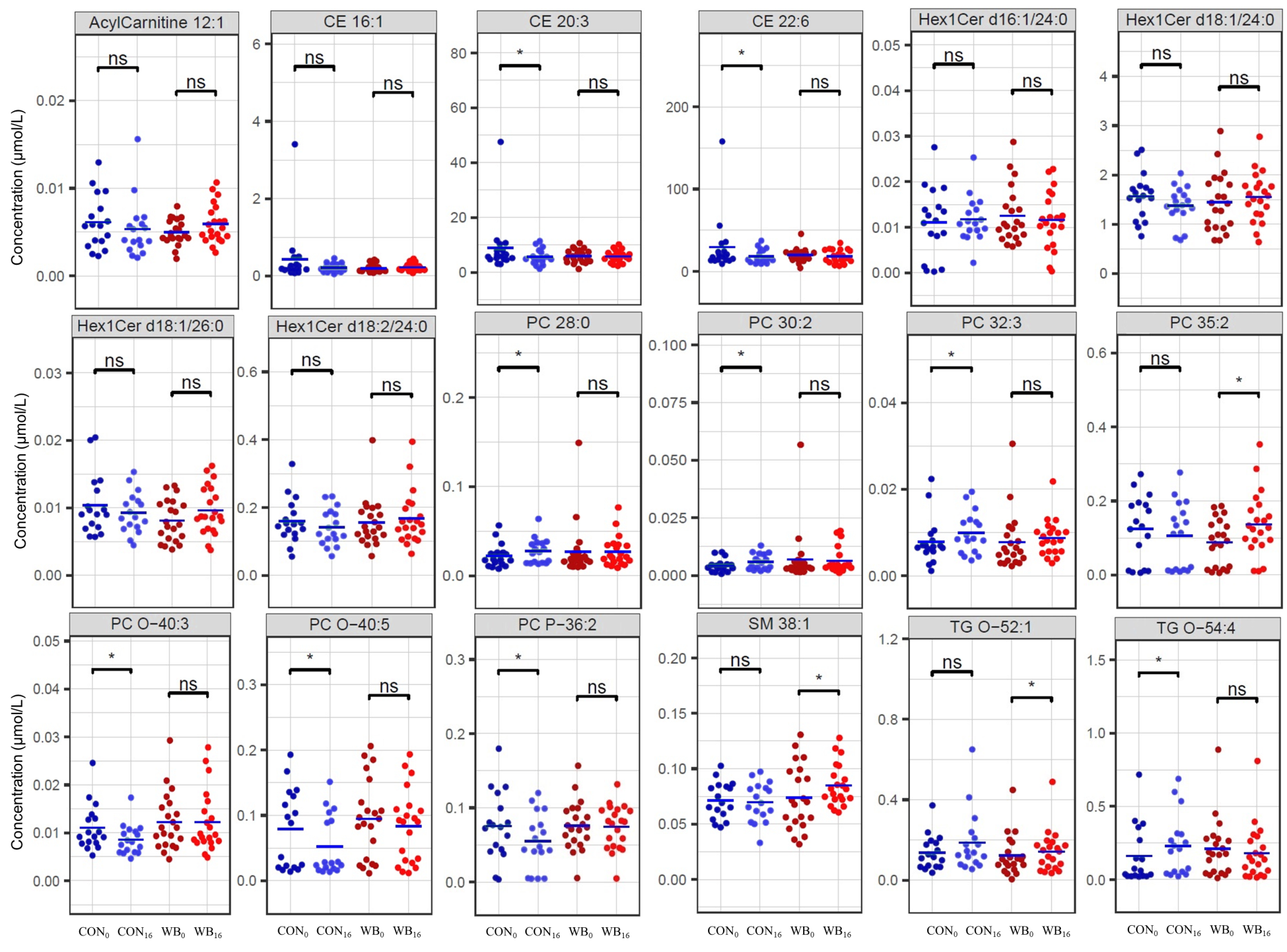


**Supplementary Figure 2** Changes to significant plasma lipidome species in the control group (●) and wolfberry group (●) at weeks 0 and 16.

* indicates significant difference with paired t-test (*P* < 0.05, > 0.2 fold difference)

CE, cholesterol ester; CON, control group; Hex1Cer, monohexosylceramide; ns, no significant difference; PC, phosphatidylcholine; PC-O, ether-phosphatidylcholine; PC-P, plasmalogen-phosphatidylcholine; SM, sphingomyelin; TG-O, ether-triglyceride; WB, wolfberry group.

**
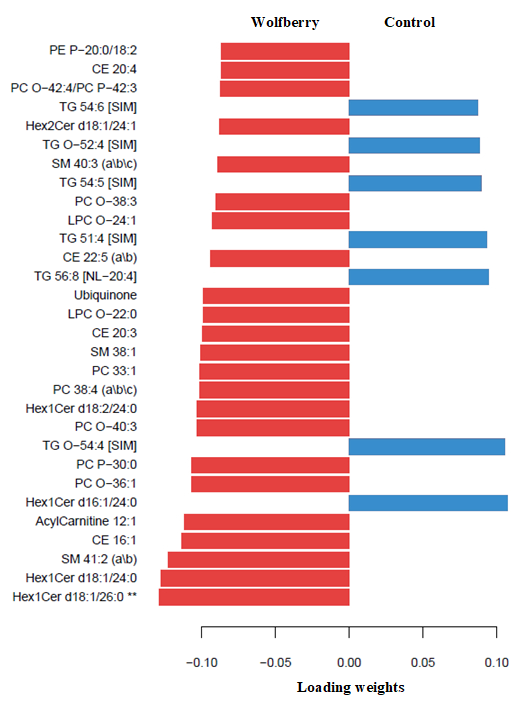
**

**Supplementary Figure 3** Loading weights for partial least squares-discriminant analysis component 1

Red: Wolfberry group; Blue: Control group

CE, cholesterol ester; Hex1Cer, monohexosylceramide; Hex2Cer, dihexosylceramide; LPC-O, ether-lysophosphatidylcholine; PC, phosphatidylcholine; PC-O, ether-phosphatidylcholine; PC-P, plasmalogen-phosphatidylcholine; PE-P, plasmalogen-phosphatidylethanolamine; SM, sphingomyelin; TG, triglyceride; TG-O, ether-triglyceride


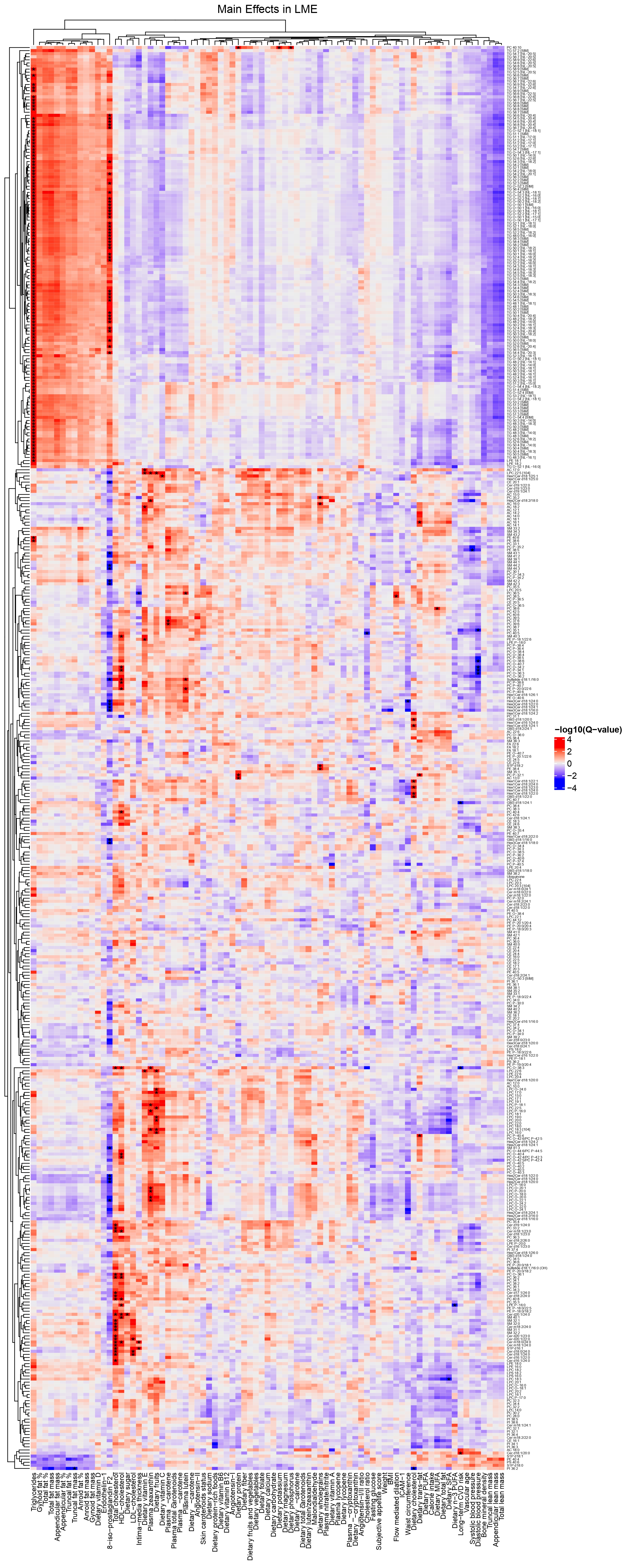


**Supplementary Figure 4** Heatmap of associations (main-effects) between lipidomic species and CVD-related outcomes and dietary/nutritional intakes

Red and blue cells represent positive and negative correlations respectively with color intensity proportional to –log_10_(*P*)


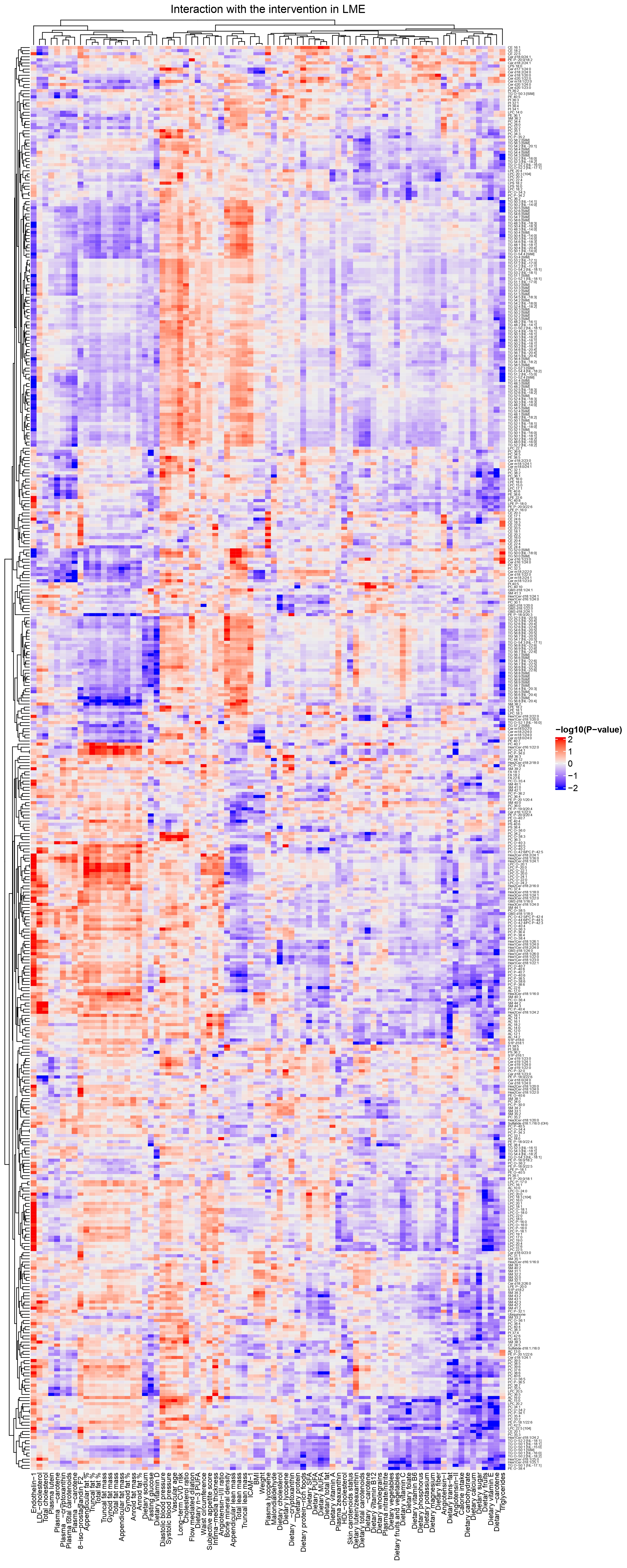


**Supplementary Figure 5** Heatmap of associations (interaction-effects) between lipidomic species and CVD-related outcomes and dietary/nutritional intakes

Red and blue cells represent positive and negative correlations respectively with color intensity proportional to –log_10_(*P*)

**Supplementary Table 2** Lipid species and clinical/dietary outcome pairs with significant main effects

| **Outcome** | **Lipid species** | **Main effect size** | **Q value** |
| --- | --- | --- | --- |
| Total cholesterol | Cer d20:1/23:0 | 0.373 | 0.0104 |
| Total cholesterol | Cer m18:0/24:0 | 0.301 | 0.0104 |
| Total cholesterol | Cer d18:1/24:0 | 0.410 | 0.0131 |
| Total cholesterol | SM 32:0 | 0.469 | 0.0170 |
| Total cholesterol | SM 32:1 | 0.441 | 0.0175 |
| Total cholesterol | Cer m18:1/24:0 | 0.408 | 0.0180 |
| Total cholesterol | PC O-36:1 | 0.490 | 0.0229 |
| Total cholesterol | PC 33:2 | 0.336 | 0.0229 |
| Total cholesterol | Cer d17:1/24:0 | 0.330 | 0.0291 |
| Total cholesterol | Cer m18:2/24:0 | 0.315 | 0.0343 |
| Total cholesterol | Cer d20:1/22:0 | 0.320 | 0.0351 |
| Total cholesterol | Cer d20:1/24:0 | 0.409 | 0.0449 |
| Total cholesterol | Cer d18:2/24:0 | 0.285 | 0.0449 |
| Total cholesterol | Cer d18:0/24:0 | 0.328 | 0.0514 |
| Total cholesterol | SM 31:1 | 0.472 | 0.0695 |
| Total cholesterol | Cer d16:1/24:0 | 0.234 | 0.0797 |
| Total cholesterol | Cer d19:1/24:0 | 0.292 | 0.1163 |
| Total cholesterol | S1P d16:1 | 0.328 | 0.1196 |
| Total cholesterol | PC 36:2 | 0.370 | 0.1312 |
| Total cholesterol | Cer m18:1/23:0 | 0.255 | 0.1522 |
| Total cholesterol | PC 40:8 | 0.308 | 0.1607 |
| Total cholesterol | PC O-38:3 | 0.266 | 0.1867 |
| LDL-cholesterol | Cer d18:0/24:0 | 0.327 | 0.0904 |
| LDL-cholesterol | Cer d18:1/24:0 | 0.332 | 0.1779 |
| LDL-cholesterol | Cer d20:1/22:0 | 0.279 | 0.1890 |
| HDL-cholesterol | PC O-38:3 | 0.357 | 0.0100 |
| HDL-cholesterol | PC 36:2 | 0.413 | 0.0345 |
| HDL-cholesterol | Cer m18:0/24:0 | 0.242 | 0.0694 |
| HDL-cholesterol | PC O-34:2 | 0.286 | 0.0695 |
| HDL-cholesterol | PC P-34:1 | 0.286 | 0.0695 |
| HDL-cholesterol | PE P-20:0/22:6 | 0.358 | 0.0747 |
| HDL-cholesterol | Cer d20:1/24:0 | 0.366 | 0.0758 |
| HDL-cholesterol | SM 40:3 | 0.455 | 0.1091 |
| HDL-cholesterol | Sulfatide d18:1:/16:0 | 0.346 | 0.1226 |
| HDL-cholesterol | PC 40:8 | 0.328 | 0.1268 |
| HDL-cholesterol | PC O-42:4/PC P-42:3 | 0.433 | 0.1312 |
| HDL-cholesterol | PC 40:4 | 0.448 | 0.1330 |
| HDL-cholesterol | LPE P-16:0 | 0.276 | 0.1370 |
| HDL-cholesterol | PC O-36:1 | 0.412 | 0.1373 |
| HDL-cholesterol | PC O-40:4 | 0.401 | 0.1567 |
| HDL-cholesterol | PC P-38:6 | 0.369 | 0.1702 |
| HDL-cholesterol | Cer m18:1/23:0 | 0.234 | 0.1722 |
| HDL-cholesterol | SM 40:1 | 0.258 | 0.1875 |
| Total cholesterol:HDL-C | PC 40:5 | -0.328 | 0.1951 |

**Supplementary Table 2** (continued)

| **Outcome** | **Lipid species** | **Main effect size** | **Q value** |
| --- | --- | --- | --- |
| Triglycerides | TG 51:4 [SIM] | 0.577 | 0.0018 |
| Triglycerides | TG O-52:4 [SIM] | 0.568 | 0.0018 |
| Triglycerides | TG 50:4 [SIM] | 0.428 | 0.0018 |
| Triglycerides | TG 50:5 [SIM] | 0.505 | 0.0018 |
| Triglycerides | TG 52:6 [NL-18:2] | 0.488 | 0.0018 |
| Triglycerides | TG 52:6 [SIM] | 0.509 | 0.0018 |
| Triglycerides | TG 53:3 [SIM] | 0.438 | 0.0021 |
| Triglycerides | TG 51:3 [SIM] | 0.419 | 0.0021 |
| Triglycerides | TG 50:4 [NL-14:0] | 0.444 | 0.0022 |
| Triglycerides | TG 50:4 [NL-18:3] | 0.434 | 0.0022 |
| Triglycerides | TG 53:2 [SIM] | 0.409 | 0.0022 |
| Triglycerides | TG 52:5 [SIM] | 0.375 | 0.0022 |
| Triglycerides | TG 51:2 [SIM] | 0.407 | 0.0027 |
| Triglycerides | TG 52:5 [NL-18:3] | 0.382 | 0.0027 |
| Triglycerides | TG 50:0 [SIM] | 0.318 | 0.0027 |
| Triglycerides | TG 48:3 [NL-16:1] | 0.499 | 0.0027 |
| Triglycerides | TG 52:0 [SIM] | 0.335 | 0.0029 |
| Triglycerides | TG 53:4 [SIM] | 0.438 | 0.0030 |
| Triglycerides | TG 51:2 [NL-15:0] | 0.605 | 0.0030 |
| Triglycerides | TG 50:0 [NL-18:0] | 0.270 | 0.0036 |
| Triglycerides | TG 53:2 [NL-18:1] | 0.402 | 0.0040 |
| Triglycerides | TG O-54:2 [NL-18:1] | 0.401 | 0.0040 |
| Triglycerides | TG 52:6 [NL-20:4] | 0.445 | 0.0040 |
| Triglycerides | TG 48:3 [NL-18:3] | 0.379 | 0.0047 |
| Triglycerides | TG O-52:3 [SIM] | 0.427 | 0.0051 |
| Triglycerides | TG 48:2 [SIM] | 0.364 | 0.0059 |
| Triglycerides | TG 50:3 [SIM] | 0.322 | 0.0064 |
| Triglycerides | TG 51:1 [SIM] | 0.314 | 0.0064 |
| Triglycerides | TG O-54:4 [NL-18:2] | 0.490 | 0.0064 |
| Triglycerides | TG 54:5 [NL-18:3] | 0.298 | 0.0066 |
| Triglycerides | TG 54:6 [NL-18:3] | 0.300 | 0.0070 |
| Triglycerides | TG 48:2 [NL-14:1] | 0.422 | 0.0070 |
| Triglycerides | TG 48:3 [NL-14:0] | 0.440 | 0.0078 |
| Triglycerides | TG 48:2 [NL-14:0] | 0.290 | 0.0084 |
| Triglycerides | TG 54:2 [NL-18:0] | 0.282 | 0.0091 |
| Triglycerides | TG 56:4 [SIM] | 0.326 | 0.0091 |
| Triglycerides | TG O-52:1 [NL-18:1] | 0.345 | 0.0091 |
| Triglycerides | TG 54:6 [SIM] | 0.263 | 0.0091 |
| Triglycerides | TG 54:2 [SIM] | 0.290 | 0.0095 |
| Triglycerides | TG 50:3 [NL-18:2] | 0.356 | 0.0097 |
| Triglycerides | TG 54:7 [SIM] | 0.367 | 0.0100 |
| Triglycerides | TG 52:2 [SIM] | 0.280 | 0.0102 |
| Triglycerides | TG 51:2 [NL-17:0] | 0.336 | 0.0102 |
| Triglycerides | TG 51:2 [NL-17:1] | 0.335 | 0.0102 |

**Supplementary Table 2** (continued)

| **Outcome** | **Lipid species** | **Main effect size** | **Q value** |
| --- | --- | --- | --- |
| Triglycerides | TG 54:3 [SIM] | 0.278 | 0.0103 |
| Triglycerides | TG 54:4 [SIM] | 0.278 | 0.0103 |
| Triglycerides | TG 51:1 [NL-17:0] | 0.294 | 0.0104 |
| Triglycerides | TG 52:1 [SIM] | 0.263 | 0.0104 |
| Triglycerides | TG 50:2 [SIM] | 0.287 | 0.0115 |
| Triglycerides | TG 52:5 [NL-20:4] | 0.308 | 0.0119 |
| Triglycerides | TG 48:2 [NL-16:1] | 0.421 | 0.0119 |
| Triglycerides | TG 50:3 [NL-14:1] | 0.380 | 0.0126 |
| Triglycerides | TG 53:2 [NL-17:1] | 0.279 | 0.0127 |
| Triglycerides | TG 52:3 [SIM] | 0.261 | 0.0132 |
| Triglycerides | TG 52:4 [SIM] | 0.267 | 0.0140 |
| Triglycerides | TG 48:1 [SIM] | 0.277 | 0.0140 |
| Triglycerides | TG 54:2 [NL-20:1] | 0.269 | 0.0140 |
| Triglycerides | TG 56:3 [SIM] | 0.280 | 0.0141 |
| Triglycerides | TG 54:5 [SIM] | 0.246 | 0.0144 |
| Triglycerides | TG 52:4 [NL-18:3] | 0.288 | 0.0149 |
| Triglycerides | TG 58:5 [SIM] | 0.262 | 0.0149 |
| Triglycerides | TG O-50:2 [NL-18:1] | 0.381 | 0.0151 |
| Triglycerides | TG 50:1 [SIM] | 0.280 | 0.0156 |
| Triglycerides | TG 50:4 [NL-20:4] | 0.258 | 0.0173 |
| Triglycerides | TG 48:1 [NL-18:1] | 0.289 | 0.0173 |
| Triglycerides | TG 50:2 [NL-16:1] | 0.303 | 0.0180 |
| Triglycerides | TG O-54:4 [SIM] | 0.380 | 0.0180 |
| Triglycerides | TG 50:3 [NL-18:3] | 0.262 | 0.0186 |
| Triglycerides | TG 48:3 [SIM] | 0.316 | 0.0186 |
| Triglycerides | TG 56:5 [SIM] | 0.349 | 0.0187 |
| Triglycerides | TG 54:3 [NL-18:2] | 0.269 | 0.0225 |
| Triglycerides | TG O-54:3 [NL-17:1] | 0.368 | 0.0229 |
| Triglycerides | TG 48:2 [NL-18:2] | 0.249 | 0.0229 |
| Triglycerides | TG 52:1 [NL-18:0] | 0.232 | 0.0229 |
| Triglycerides | TG 50:1 [NL-14:0] | 0.257 | 0.0229 |
| Triglycerides | TG 52:1 [NL-18:1] | 0.231 | 0.0236 |
| Triglycerides | TG 50:3 [NL-14:0] | 0.311 | 0.0249 |
| Triglycerides | TG 54:4 [NL-20:3] | 0.488 | 0.0264 |
| Triglycerides | TG 50:2 [NL-14:0] | 0.357 | 0.0279 |
| Triglycerides | LPE 18:1 | 0.381 | 0.0289 |
| Triglycerides | TG 54:6 [NL-20:4] | 0.262 | 0.0291 |
| Triglycerides | TG 48:0 [NL-16:0] | 0.237 | 0.0303 |
| Triglycerides | TG 52:2 [NL-18:2] | 0.219 | 0.0313 |
| Triglycerides | TG 54:4 [NL-18:2] | 0.255 | 0.0313 |
| Triglycerides | TG 52:2 [NL-16:0] | 0.256 | 0.0324 |
| Triglycerides | TG 56:8 [SIM] | 0.353 | 0.0324 |
| Triglycerides | PE 40:6 | 0.597 | 0.0329 |

**Supplementary Table 2** (continued)

| **Outcome** | **Lipid species** | **Main effect size** | **Q value** |
| --- | --- | --- | --- |
| Systolic blood pressure | PE 38:5 | -0.338 | 0.1782 |
| Diastolic blood pressure | PC 35:1 | -0.496 | 0.0512 |
| Diastolic blood pressure | PC O-36:3 | -0.470 | 0.1268 |
| Diastolic blood pressure | PC O-34:2 | -0.366 | 0.1343 |
| Diastolic blood pressure | PC P-34:1 | -0.366 | 0.1343 |
| Diastolic blood pressure | PC O-38:6 | -0.428 | 0.1542 |
| Long-term CVD risk | PC O-38:3 | -0.346 | 0.0174 |
| Long-term CVD risk | Cer d18:1/20:0 | 0.238 | 0.0848 |
| Long-term CVD risk | GM3 d18:1/24:1 | -0.352 | 0.1036 |
| Flow mediated dilation | PC 38:5 | 0.532 | 0.1951 |
| Intima-media thickness (right) | PC 36:5 | -0.336 | 0.0291 |
| Intima-media thickness (right) | CE 20:5 | -0.392 | 0.0996 |
| Intima-media thickness (right) | Cer m18:0/24:0 | 0.241 | 0.1267 |
| Intima-media thickness (right) | PC O-36:5 | -0.207 | 0.1725 |
| Intima-media thickness | S1P d16:1 | 0.287 | 0.0449 |
| Intima-media thickness | PC 36:5 | -0.225 | 0.1546 |
| Intima-media thickness | Cer m18:0/24:0 | 0.179 | 0.1867 |
| Waist circumference | Hex3Cer d18:1/24:0 | -0.268 | 0.1681 |
| Truncal fat mass | PI 36:2 | -0.218 | 0.1379 |
| Plasma 8-isoprostanes | Hex3Cer d18:1/16:0 | -0.662 | 0.0092 |
| Plasma 8-isoprostanes | TG 54:6 [NL-20:4] | 0.576 | 0.0115 |
| Plasma 8-isoprostanes | Hex3Cer d18:1/24:1 | -0.610 | 0.0162 |
| Plasma 8-isoprostanes | SM 42:3 | -0.632 | 0.0193 |
| Plasma 8-isoprostanes | TG 56:8 [NL-20:4] | 0.520 | 0.0238 |
| Plasma 8-isoprostanes | TG 56:7 [NL-20:4] | 0.530 | 0.0324 |
| Plasma 8-isoprostanes | Hex3Cer d18:1/22:0 | -0.620 | 0.0362 |
| Plasma 8-isoprostanes | TG 50:4 [NL-20:4] | 0.499 | 0.0484 |
| Plasma 8-isoprostanes | SM 42:2 | -0.643 | 0.0533 |
| Plasma 8-isoprostanes | Hex2Cer d18:1/22:0 | -0.723 | 0.0616 |
| Plasma 8-isoprostanes | TG 56:6 [NL-20:4] | 0.519 | 0.0674 |
| Plasma 8-isoprostanes | Hex3Cer d18:1/18:0 | -0.516 | 0.0683 |
| Plasma 8-isoprostanes | GM3 d18:1/16:0 | -0.644 | 0.0695 |
| Plasma 8-isoprostanes | TG 54:5 [NL-20:4] | 0.513 | 0.0711 |
| Plasma 8-isoprostanes | TG 54:5 [SIM] | 0.451 | 0.0761 |
| Plasma 8-isoprostanes | TG 48:2 [NL-18:2] | 0.463 | 0.0852 |
| Plasma 8-isoprostanes | TG 52:5 [NL-20:4] | 0.500 | 0.0903 |
| Plasma 8-isoprostanes | TG 50:1 [NL-16:0] | 0.458 | 0.0906 |
| Plasma 8-isoprostanes | TG 52:6 [NL-20:4] | 0.567 | 0.0913 |
| Plasma 8-isoprostanes | TG 52:2 [NL-18:2] | 0.415 | 0.0919 |
| Plasma 8-isoprostanes | TG 50:2 [NL-18:2] | 0.429 | 0.0932 |
| Plasma 8-isoprostanes | TG 48:0 [NL-16:0] | 0.436 | 0.0935 |
| Plasma 8-isoprostanes | SM 44:3 | -0.598 | 0.0980 |
| Plasma 8-isoprostanes | TG 54:6 [SIM] | 0.431 | 0.0988 |
| Plasma 8-isoprostanes | TG 50:0 [NL-18:0] | 0.456 | 0.1009 |
| Plasma 8-isoprostanes | CE 20:1 | -0.587 | 0.1020 |
| Plasma 8-isoprostanes | TG 52:4 [SIM] | 0.456 | 0.1024 |

**Supplementary Table 2** (continued)

| **Outcome** | **Lipid species** | **Main effect size** | **Q value** |
| --- | --- | --- | --- |
| Plasma 8-isoprostanes | TG 52:4 [NL-18:3] | 0.483 | 0.1025 |
| Plasma 8-isoprostanes | TG 52:4 [NL-18:2] | 0.477 | 0.1237 |
| Plasma 8-isoprostanes | TG 52:3 [NL-18:2] | 0.456 | 0.1238 |
| Plasma 8-isoprostanes | TG 58:4 [SIM] | 0.391 | 0.1238 |
| Plasma 8-isoprostanes | TG 58:2 [SIM] | 0.388 | 0.1267 |
| Plasma 8-isoprostanes | TG O-50:1 [NL-15:0] | 0.407 | 0.1268 |
| Plasma 8-isoprostanes | TG 52:1 [NL-18:0] | 0.405 | 0.1268 |
| Plasma 8-isoprostanes | TG 50:1 [SIM] | 0.451 | 0.1302 |
| Plasma 8-isoprostanes | TG O-52:2 [NL-17:1] | 0.391 | 0.1312 |
| Plasma 8-isoprostanes | Hex2Cer d18:1/24:0 | -0.701 | 0.1312 |
| Plasma 8-isoprostanes | TG O-50:1 [NL-18:1] | 0.384 | 0.1370 |
| Plasma 8-isoprostanes | TG 48:2 [NL-14:0] | 0.457 | 0.1379 |
| Plasma 8-isoprostanes | SM 44:2 | -0.571 | 0.1410 |
| Plasma 8-isoprostanes | Hex3Cer d18:1/24:0 | -0.573 | 0.1428 |
| Plasma 8-isoprostanes | TG 58:3 [SIM] | 0.653 | 0.1433 |
| Plasma 8-isoprostanes | TG 50:3 [NL-18:3] | 0.447 | 0.1452 |
| Plasma 8-isoprostanes | TG 52:1 [NL-18:1] | 0.398 | 0.1466 |
| Plasma 8-isoprostanes | TG 58:5 [SIM] | 0.424 | 0.1478 |
| Plasma 8-isoprostanes | SM 43:1 | -0.596 | 0.1492 |
| Plasma 8-isoprostanes | SM 41:3 | -0.490 | 0.1519 |
| Plasma 8-isoprostanes | LPC O-22:1 | -0.449 | 0.1721 |
| Plasma 8-isoprostanes | Sulfatide d18:1:/16:0 | -0.464 | 0.1722 |
| Plasma 8-isoprostanes | TG 52:3 [SIM] | 0.408 | 0.1742 |
| Plasma 8-isoprostanes | TG O-54:3 [NL-18:1] | 0.385 | 0.1779 |
| Plasma 8-isoprostanes | TG O-50:2 [NL-18:2] | 0.378 | 0.1779 |
| Plasma 8-isoprostanes | TG O-52:2 [NL-18:1] | 0.382 | 0.1853 |
| Plasma 8-isoprostanes | TG O-50:1 [SIM] | 0.372 | 0.1853 |
| Plasma 8-isoprostanes | TG 54:4 [NL-20:3] | 0.634 | 0.1855 |
| Plasma 8-isoprostanes | TG O-50:1 [NL-16:0] | 0.374 | 0.1886 |
| Plasma 8-isoprostanes | TG 54:2 [NL-20:1] | 0.395 | 0.1951 |
| Plasma 8-isoprostanes | Hex2Cer d18:1/20:0 | -0.542 | 0.1957 |
| Plasma 8-isoprostanes | TG 54:3 [NL-18:2] | 0.419 | 0.1976 |
| Plasma α-carotene | PC 37:6 | 0.575 | 0.0362 |
| Plasma α-carotene | PC 39:6 | 0.511 | 0.1088 |
| Plasma lutein | PC 36:5 | 0.698 | 0.1238 |
| Plasma lutein | Sulfatide d18:1:/16:0 | 0.464 | 0.1466 |
| Plasma lutein | PE P-20:0/22:6 | 0.498 | 0.1466 |
| Plasma zeaxanthin | LPC O-20:1 | 0.461 | 0.0587 |
| Plasma zeaxanthin | LPC P-20:0 | 0.461 | 0.0587 |
| Plasma zeaxanthin | LPC O-22:1 | 0.435 | 0.1091 |
| Plasma zeaxanthin | LPC P-16:0 | 0.492 | 0.1330 |
| Plasma zeaxanthin | LPC P-18:1 | 0.471 | 0.1517 |
| Plasma zeaxanthin | Hex2Cer d18:2/18:0 | 0.451 | 0.1522 |
| Plasma zeaxanthin | LPC 22:5 [104] | 0.713 | 0.1533 |
| Plasma zeaxanthin | PC O-38:3 | 0.404 | 0.1779 |

**Supplementary Table 2** (continued)

| **Outcome** | **Lipid species** | **Main effect size** | **Q value** |
| --- | --- | --- | --- |
| Plasma zeaxanthin | LPC O-20:0 | 0.401 | 0.1951 |
| Plasma zeaxanthin | LPC 18:3 [104] | 0.482 | 0.1957 |
| Dietary protein | PC 40:10 | 0.755 | 0.0006 |
| Dietary protein | PC P-32:1 | 0.499 | 0.1652 |
| Dietary protein | AC 13:0 | 0.769 | 0.1859 |
| Dietary saturated fat | LPC 18:3 | -0.702 | 0.1247 |
| Dietary monounsaturated fat | PC 38:6 | 0.687 | 0.1516 |
| Dietary n-3 polyunsaturated fat | LPE P-16:0 | -0.500 | 0.1632 |
| Dietary trans-fat | PC P-40:4 | 0.563 | 0.0761 |
| Dietary trans-fat | AC 16:1 | 0.699 | 0.1330 |
| Dietary trans-fat | PC P-32:1 | 0.500 | 0.1976 |
| Dietary cholesterol | Hex1Cer d18:1/22:0 | 0.689 | 0.0121 |
| Dietary cholesterol | GM3 d18:1/22:0 | 0.624 | 0.0326 |
| Dietary cholesterol | GM3 d18:1/20:0 | 0.564 | 0.0613 |
| Dietary cholesterol | Hex1Cer d18:1/23:0 | 0.557 | 0.0689 |
| Dietary cholesterol | PI 36:2 | -0.670 | 0.0711 |
| Dietary cholesterol | Hex1Cer d18:1/22:1 | 0.623 | 0.0832 |
| Dietary cholesterol | Hex1Cer d18:1/24:1 | 0.585 | 0.1517 |
| Dietary vitamin C | Hex1Cer d18:1/25:1 | 0.599 | 0.1872 |
| Dietary vitamin E | LPC 22:5 [104] | 0.916 | 0.0587 |
| Dietary vitamin E | PE P-18:1/22:6 | 0.561 | 0.0859 |
| Dietary vitamin E | LPC 22:6 | 0.861 | 0.0878 |
| Dietary vitamin E | AC 17:0 | 0.541 | 0.1373 |
| Dietary vitamin E | AC 18:2 | 0.571 | 0.1571 |
| Dietary potassium | PC 40:10 | 0.477 | 0.0925 |
| Dietary phosphorus | PC 40:10 | 0.503 | 0.1025 |
| Dietary wholegrain servings | PC 35:2 | 0.554 | 0.0988 |
| Dietary wholegrain servings | AC 16:0 | 0.603 | 0.1330 |
| Dietary wholegrain servings | S1P d18:2 | 0.462 | 0.1533 |
| Dietary wholegrain servings | PE 38:4 | 0.434 | 0.1821 |
| Dietary fruit servings | LPC 22:5 [104] | 0.914 | 0.0338 |
| Dietary fruit servings | LPC 18:3 [104] | 0.584 | 0.0799 |
| Dietary fruit servings | LPC 19:0 | 0.612 | 0.0848 |
| Dietary fruit servings | LPC 22:5 | 0.725 | 0.1155 |
| Dietary fruit servings | LPC 20:0 | 0.879 | 0.1193 |
| Dietary fruit servings | LPC 17:0 | 0.576 | 0.1287 |
| Dietary fruit servings | LPC O-24:0 | 0.529 | 0.1538 |
| Dietary fruit servings | LPC 16:0 | 0.574 | 0.1538 |
| Dietary fruit servings | LPC 22:6 | 0.788 | 0.1600 |
